# Prevalence of Respiratory Manifestations in Chronic Kidney Diseases; A Descriptive Cross-sectional Study in a Tertiary Care Hospital of Nepal

**DOI:** 10.31729/jnma.4284

**Published:** 2019-04-30

**Authors:** Pankaj Pant, Santosh Baniya, Ashish Jha

**Affiliations:** 1 Department of Pulmonology and Critical care, Institute of Medicine, Maharajgunj, Nepal; 2 Department of General Practice and Emergency Medicine, Institute of Medicine, Maharajgunj, Nepal; 3 Department of Medicine, Institute of Medicine, Maharajgunj, Nepal

**Keywords:** *Chronic kidney diseases*, *obstructive sleep apnoea*, *pulmonary oedema*, *pulmonary tuberculosis*, *respiratory manifestations.*

## Abstract

**Introduction:**

Chronic kidney diseases affect patients with multiple respiratory complications by varied etiopathogenesis adversely affecting the outcome in them. The aim of the study is to find out the prevalence of respiratory manifestations among patients with chronic kidney disease.

**Methods:**

The descriptive cross-sectional study was carried out tertiary care hospital from January 2019 to March 2019 after ethical approval. One hundred and sixty five patients with established chronic kidney diseases being treated in a tertiary hospital for a month were included for the study. Clinical evaluation and relevant investigations; chest xray, pleural fluid analysis, sputum analysis, echocardiography, biochemical investigations and hematological investigations were done to assess the respiratory manifestations of the patients. Statistical Package for Social Sciences version 22 was used for the analysis of the data and point estimate at 95% Confidence interval was calculated along with frequency and proportion for binary data and the analysis was done.

**Results:**

The prevalence of respiratory manifestations was 102 (61.8%) at 95% Confidence interval, range occurring between 55% to 69%. Pulmonary oedema 41 (24.84%) was the most common manifestation followed by pleural effusion 18 (10.9%). Pleural effusions were predominantly bilateral and transudative type. Pneumonia 17 (10.3%) was predominantly lobar pneumonia. Sixteen (9.7%) of the patients were screened positive for obstructive sleep apnoea syndrome. Pulmonary tuberculosis was present in 9 (5.45%) patients.

**Conclusions:**

Varieties of respiratory complications can present in varied spectrum in patients with chronic kidney diseases and this carries adverse outcome to patient management as well as affects the quality of life of patient and their family.

## INTRODUCTION

Globally, patients living with stage V chronic kidney disease (CKD) and receiving renal replacement therapy is estimated to be over 1.4 million and this is increasing by 8% annually.^1^ Chronic kidney disease is found to increase the risk of cardiovascular diseases, premature deaths and decreased quality of life resulting from systemic complications^2^ and respiratory complications are found to be commonly associated in these patients.^3^ The patho-physiological explanation of respiratory complications is the result of alteration in volume status in Chronic kidney disease patients, altered immune status and concomitant heart failure although the exact mechanism is not well understood.^4^

Despite respiratory complications being common in these patients there is no statistical figures to point out the prevalence of such illnesses and their burden in CKD patients in Nepalese context. Hence, this study was conducted in Tribhuvan University Teaching Hospital, Maharajgunj, and Kathmandu.

Hence, the aim of the study is to find out the prevalence of respiratory manifestations among patients with chronic kidney disease.

## METHODS

This study was descriptive cross-sectional study conducted in Tribhuvan University Teaching Hospital among in-patients who had established chronic kidney disease as per Kidney diseases improving global outcomes (KDIGO) criteria and was admitted for respiratory complications from January 15 to February 15, 2019. Prior ethical approval was taken from the Institutional review board (IRB) Reference no 335 (6–11) E^2^ 075/76 of Institute of Medicine and informed consents were taken from the participants. Study population were the patients admitted to medical ward of a tertiary care hospital. Total of 165 patients were enrolled in the study by convenience sampling. The inclusion criteria for the study was Patients (>16 years) with established chronic kidney diseases stage 3, 4 and 5 as per KDIGO guideline presenting with respiratory illnesses who provide written consent for the study and the exclusion criteria were patients less than 16 years, acute kidney injury, presenting symptoms other than respiratory illness, renal allograft patients and co- morbidities like HIV, chronic liver diseases.

Sample size was calculated by using the formula given below:


$$\begin{array}{l} \text{Sample size (n)}={\text{Z}}^{2}\times \text{p}{\text{(1-p)/e}}^{2}\\ =1.96^{2}\times 0.7\text{​}\times (0.3)/{\left(0.07\right)}^{2}\\ =165 \end{array}$$

Where,

Z= 1.96, value at confidence interval 95%>
p= prevalence of study, 70% (educated guess)
e= margin of error, 7%>

Hence, the sample size for the study was calculated and found to be 165.

Selection and information bias has been minimized as possible.

Data were collected in socio-demographic profile, caused of CKD with duration of illness, haematological parameters, biochemical parameters and radiological parameters and filled in pre designed proforma.

SPSS version 22 was used for the analysis of the data. Point estimate at 95% Confidence interval (CI) was calculated along with frequency and proportion for binary data and the analysis was done.

## RESULTS

Hence, the prevalence of respiratory manifestations was 102 (61.8%) at 95% confidence interval, range occurring between 55% to 69%.

Cough was the most common respiratory symptom 66 (40%) followed by shortness of breath 65 (39.4%). The prevalence of other presenting symptoms is as follows:

Respiratory disease in the patients studied: Pulmonary Edema was the most common respiratory manifestation 41 (24.84%), followed by pleural effusion 18 (10.9%) and pneumonia 17 (10.3%) among the patients presenting to the hospital. The prevalence of other respiratory manifestations is as follows:

Out of 18 them, only 13 effusions were amenable to pleural tapping and the study of the pleural fluid showed that 10 were transudative and 3 were exudative pleural effusion as per the Light's criteria. The remaining 5 non amenable effusions were associated with consolidation. Stratification of the pleural effusion according to the CKD stage (Table 3).

**Table 1:** Prevalence of symptoms of patients presenting with respiratory illness.

Symptoms	n (%)
Cough	66 (40)
Shortness of breath	65 (39.4)
Decreased appetite	43 (26.06)
Fever	41 (24.84)
Weight loss	34 (20.6)
PND	25 (15.15)
Chest pain	24 (14.14)
Difficulty sleeping	16 (9.7)
Orthopnea	10 (6.06)
Hemoptysis	7 (4.24)

**Table 2:** Prevalence of respiratory manifestations in the studied patients

Diagnosis	n (%)
Pulmonary Edema	41 (24.84)
Pleural Effusion	18 (10.9)
Pneumonia	17 (10.3)
Obstructive Sleep Apnoea Syndrome	16 (9.7)
Pulmonary Tuberculosis	9 (5.45)
Acute Exacerbation of Interstitial Lung Disease	1 (0.60)

**Table 3:** Prevalence of respiratory manifestations according to CKD stages.

Common Pulmonary Diseases as per Stage of CKD				
	Stage			Total
	III n (%)	IV n (%)	V n (%)	
Pulmonary Edema	3 (1.8)	7 (4.2)	31 (18.7)	41
Pleural Effusion	0	3 (1.8)	15 (9.09)	18
Pneumonia	0	3 (1.8)	14 (8.4)	17
Pulmonary TB	2 (1.2)	1 (0.6)	6 (3.6)	9

Our study showed 17 (10.3%) patients had pneumonia. 14 patients had lobar pneumonia and 3 patients had bronchopneumonia. Out of the 17 patients, 5 patients had associated pleural effusion.

Obstructive sleep apnea was screened in all the patients using "STOP BANG" questionnaire and it was positive in 16 (9.7%) patients. Due to the unavailability of polysomnography in the TUTH diagnosis could not be confirmed.

Tuberculosis was present in 14 patients who comprised of 9 (5.45%) pulmonary tuberculosis and 5 extra pulmonary tuberculosis. Among the 9 patients with pulmonary tuberculosis, 3 patients had cavitations on radiological evaluation and all of them were sputum positive for acid fast bacilli. All the patients with pulmonary TB had cough and weight loss as presenting complaints while fever was present only in 5 patients.

## DISCUSSION

Based on the fact that the respiratory illnesses are common culprit in increasing the hospital admissions among patients living with chronic kidney disease and resulting in the decreased quality of life we conducted this study in one of the tertiary hospitals of Nepal. And, the results have clearly shown that a variety of respiratory manifestations are prevalent in Nepalese context. In addition, the results also lights up the fact that these complications should not be overlooked. In fact, meticulous analysis and work up might be needed in some situations.

Acute pulmonary edema is most common cause of hospital admission in chronic kidney disease patients especially in patients with end stage renal disease.^5^ In fact; pulmonary edema is one of the leading causes of increased mortality and morbidity among chronic kidney disease patients. Pulmonary edema was the most common respiratory manifestation in CKD patients 41 (24.84%) in our study and patients presented with chief complaints of cough and shortness of breath. However, only a few of our patients presented with orthopnea and paroxysmal nocturnal dyspnea.

The mechanism of pulmonary edema is multifactorial. Decrease in the plasma oncotic pressure as a result of hypoalbuminemia is a characteristic finding in chronic kidney disease along with the increase in the hydrostatic pressure as a result of volume overload and left ventricular diastolic dysfunction.^3,4^ These factors results in fluids to leak through the pulmonary capillaries leading to the development of pulmonary edema. Similarly, there is alteration in pulmonary capillary permeability evidenced in the euvolemic patients.^3^ In our study 65 patients had evidence of left ventricular diastolic dysfunction and 28 off them developed pulmonary edema. All of the patients with the pulmonary edema didn't undergo echocardiography due to economical constrains. Similarly, mean serum albumin level was 31.13±5.59 g/l which ranged from 20 to 53 g/l.

Pleural effusion is a common finding in the chronic kidney disease patients^1^ and is second most common respiratory manifestation in our study. The effusion was predominantly bilateral and transudative.

The etiopathogenesis of pleural effusion is also multifactorial. In addition to uremic pleuritis, over hydration, left ventricular diastolic dysfunction, pulmonary infection, hypoalbuminemia are important contributing factor in developing the pleural effusion.^6^ Pleural effusion secondary to over hydration and heart failure are of bilateral and transudative type and effusion secondary to uremic pleuritis and pulmonary infection are of unilateral and exudative type.

Echocardiography finding of left ventricular diastolic dysfunction was very common among the patients in our study.

Pneumonia was another common respiratory manifestation in our studied patients and majority were lobar pneumonia. In patients with chronic kidney disease, pneumonia is the most common infectious co-morbidity.^7^ A comparative longitudinal study has shown that the incidence density rate of pneumonia was significantly greater among patients with chronic kidney diseases compared to general population in both inpatient and outpatient setting.^7^ This study also concluded that co-morbidities like diabetes mellitus, cardiovascular disease, asthma and chronic obstructive pulmonary diseases were independent associated risk factor for pneumonia.

Despite obstructive sleep apnea syndrome (OSAS) being another common manifestation, due to limitation in performing diagnostic tests, we cannot conclude the prevalence and provide recommendation. However, the screening test in our study clearly demonstrates that OSAS likely is a common manifestation.

Studies have shown that sleep apnea is very common in chronic kidney patients^8,9^ and these studies recommends to look for other conditions like restless leg syndrome and periodic limb movement disorder. Despite several proposed mechanisms, the mechanism is still unknown. In addition, it is found that undergoing hemodialysis during night has ameliorating effect in sleep apnea.^8,10^ And, intermittent positive pressure ventilation is effective for treatment.^10,11^

In comparison to general population the prevalence of both pulmonary and extra pulmonary tuberculosis in chronic kidney disease patients is high.

Chronic kidney disease is associated with altered cellular immune status which is affected by various factors like advanced age, malnutrition, hypoalbuminemia, uraemia and medical immunosupperssion.^12^ In addition, patients frequently visit hospitals and dialysis centres for the management and increase the risk of developing tuberculosis.^12^ The incidence of tuberculosis in chronic kidney disease patients is6 to 16 times more in comparison to general population.^13^ Meanwhile, one study showed the incidence of 4% with predominance of extra pulmonary tuberculosis compared to pulmonary TB.^14^

## CONCLUSIONS

This study concludes that pulmonary edema is the most common respiratory manifestation in chronic kidney disease patients and this is followed by pleural effusion and pneumonia.

Pulmonary and extra pulmonary tuberculosis are also common presentation and meticulous workup should be done in patients with high clinical suspicion. Obstructive sleep apnea syndrome might be an emerging complication in our part of the world and careful workup is necessary in patients who are screened positive for obstructive sleep apnoea.

## Conflict of Interest

**None.**
